# *Chitin Deacetylase 1* Gene as an Optimal RNAi-Based Target for Controlling the Tomato Leaf Miner *Tuta absoluta*

**DOI:** 10.3390/insects15110838

**Published:** 2024-10-25

**Authors:** Yangfan Zhou, Yu Zhang, Kangkang Xu, Ruiyu Liu, Wenbiao Liu, Hang Ma, Wenjia Yang

**Affiliations:** 1 Key Laboratory of Surveillance and Management of Invasive Alien Species in Guizhou Education Department, College of Biological and Environmental Engineering, Guiyang University, Guiyang 550005, China; pingjiaboys@163.com (Y.Z.); zy16789421069@163.com (Y.Z.); sabc20040206@163.com (R.L.); yangwenjia10@126.com (W.Y.); 2 Yunnan Yuantianhua Co., Ltd. Research and Development Center, Kunming 650228, China; abiaohao2022@163.com

**Keywords:** chitin deacetylase, RNA interference, *Tuta absoluta*, molting and metamorphosis

## Abstract

**Simple Summary:**

The tomato leaf miner, *Tuta absoluta*, is an invasive pest that causes serious losses in tomato cultivation worldwide. In this study, a *chitin deacetylase 1* gene (*TaCDA1*) was identified and characterized as a potential RNAi target to control *T. absoluta*. The complete open reading frame of *TaCDA1* was cloned and sequence information was annotated. The protein encoded by *TaCDA1* contained conserved domains of a typical class I CDA (chitin deacetylase) protein and had high homology with insects from Lepidoptera. qPCR showed that 5 d old pupae had the highest *TaCDA1* expression level during development, and this gene was more highly expressed in the epidermis than in other tissues. dsRNA targeting *TaCDA1* was synthesized and injected into pupae and larvae. Successful downregulation of *TaCDA1* was detected, and high mortality was observed during the pupal–adult and larval–pupal transition. The phenotypes caused by RNAi of *TaCDA1* included abnormal epidermis formation and weight loss, which was consistent with the predicted function and expression characteristics of *TaCDA1*. Oral delivery of dsRNA at the larval stage was also tested, but no effects were observed.

**Abstract:**

Chitin is a critical component of both the exoskeleton and internal structures of insects, which can protect insects from mechanical damage, dehydration and pathogen infection, and plays a significant role in the molting process. Chitin deacetylases (CDAs), key enzymes involved in chitin metabolism, are widely distributed among arthropods and microorganisms. In this study, we identified a *CDA* gene, *TaCDA1*, in the invasive insect species *Tuta absoluta* (Meyrick). Sequence analysis demonstrated a high degree of similarity to CDAs in other insects, revealing the presence of three conserved domains. Quantitative analysis showed that the *TaCDA1* gene exhibited peak expression during the pupal stage, particularly within the epidermis. The suppression of *TaCDA1* expression through RNA interference in *T. absoluta* pupae significantly impacted the expression of genes associated with chitin metabolism, increasing mortality and developmental abnormalities during the pupa–adult transition and reducing the pupal weight. Furthermore, soaking gene-specific dsRNA resulted in elevated mortality rates during the larva–pupa transition, causing the inability to form new cuticles or undergo ecdysis, as confirmed by subsequent histological observations. The oral administration of ds*TaCDA1* + sucrose solution did not significantly impact *NtCDA1* expression or the mortality rate compared to the ds*GFP* + sucrose solution control in the non-target insect *Nesidiocoris tenuis*. This study demonstrated that *TaCDA1* is a potential and safe target for pest control of *T. absoluta*.

## 1. Introduction

Chitin, a linear polysaccharide polymer consisting of many *N*-acetylglucosamine units linked by *β*-1,4 glycosidic bonds, is a major component of the arthropod exoskeleton [1]. Chitin is found in the exoskeleton and internal structures of insects and plays an indispensable role in the molting process, enabling insects to change their exoskeletons regularly to adapt during different growth stages [2]. Chitin deacetylases (CDAs, EC 3.5.1.41), key enzymes involved in chitin metabolism, are widely found in arthropods and microorganisms. CDAs belong to family 4 of the carbohydrate esterases (CE-4), which convert chitin to chitosan through *N*-deacetylation [3]. CDAs have been classified into five groups (Groups I–V) according to their sequence homology and domain diversity, and all CDAs have polysaccharide deacetylase-like catalytic domains (4CE-domain). Groups I (CDA1 and CDA2) and II (CDA3) also have a chitin-binding peritrophin-A domain (ChBD) and a low-density lipoprotein receptor class A domain (LDLa) [4]. Among insect CDAs, CDA1 has been reported to have abundant functions. The role of CDA1 has been well studied as an essential function in insect larval–larval, larval–pupal, and pupal–adult molting of *Sogatella furcifera* [5], *Heortia vitessoides* [6], *Tribolium castaneum* [7], *Nilaparvata lugens* [8], *Stegobium paniceum* [9], *Leptinotarsa decemlineata* [10], and *Holotrichia parallela* [11]. CDA1 has also been found to affect insect wing development [12,13]. Subsequent studies have shown that CDA1 protects against bacterial and fungal infections through deacetylation from chitin [14,15,16]. Recently, the role of insect CDA1 in pesticide resistance has also been identified [17].

RNA interference (RNAi) is a promising and widely used tool for pest control that has the advantages of being simple, efficient, and specific [18]. Pest control with RNAi relies on silencing specific genes for reproduction, growth, or development in the insect pest [19]. However, the RNAi efficiency of Lepidoptera insects is very poor and greatly limits the use of RNAi-based pest control strategies [20]. Studies have recently shown that the RNAi strategy for Lepidoptera insects can be effectively improved by identifying effective functional genes. In *Manduca sexta*, silencing the Ecdysis-triggering hormone and its receptor by injecting dsRNA into the ventral abdomen of females delayed egg laying and significantly reduced egg production [21]. *Chilo partellus* larvae exhibited various phenotypic distortion levels across developmental stages, and 53% of larvae died after silencing *chitinase* genes in transgenic maize lines expressing dsRNA [22]. In *Tuta absoluta*, dsRNAs targeting the juvenile hormone inducible protein, juvenile hormone epoxide hydrolase protein, ecdysteroid 25-hydroxylase, chitin synthase A, carboxylesterase, and arginine kinase were expressed in *Escherichia coli* HT115 (DE3), increasing larval mortality and seriously impacting larval development [23]. In *Helicoverpa zea* and *Heliothis virescens*, larvae were fed an artificial diet containing the dsRNA target-silencing pheromone biosynthesis-activating neuropeptide, delaying larval growth, interfering with pupal development, and causing mortality in these two pest moths [24].

The tomato leaf miner, *Tuta absoluta* (Meyrick) (Lepidoptera: Gelechiidae), is one of the most destructive invasive pests and has now invaded more than 100 countries and regions [25]. It is a major threat to tomato cultivation worldwide and causes heavy losses in tomato production when management strategies are not efficient [26]. Chemical pest control methods are still the most commonly used method for controlling *T. absoluta*. However, the overuse of hazardous chemicals has caused high resistance in *T. absoluta* and serious health issues in the environment, non-targeted animals, and humans [27]. Therefore, the development of a green control strategy for *T. absoluta* is of practical importance. Chitin is essential for insects and absent in vertebrates; thus, chitin deacetylases are efficient and safe RNAi targets for pest control. In this study, we identified *CDA1* gene in *T. absoluta* and analyzed *CDA1* expression at different developmental stages and in different tissues. Furthermore, we estimated the pest control potential of *CDA1* and tested its effects on the natural enemy (*Nesidiocoris tenuis*) to provide an effective and safe target for an RNAi-based pest control strategy for *T. absoluta*.

## 2. Materials and Methods

### 2.1. Experimental Insects

The tomato leaf miner population utilized in this study was obtained in 2023 from Kunming City, Yunnan Province, China. The laboratory conditions were maintained as follows: temperature, 26 ± 1 °C; photoperiod, 16 L: 8 D; relative humidity, 60 ± 5%. The larvae were fed fresh tomato leaves, while the adults were offered 10% honey water.

### 2.2. cDNA Cloning and Bioinformatics Analysis of TaCDA1

The total RNA of the 4th-instar larva of *T. absoluta* was isolated using a MiniBEST Universal Extraction Kit (Takara, Dalian, China), and first-strand cDNA synthesis was accomplished using 1 μg of total RNA and a SMART^®^ MMLV Reverse Transcriptase Kit (Takara). Based on the transcriptome database (SRR13065833) of *T. absoluta*, the specific amplification primers for the open reading frame (ORF) of *TaCDA1* were used in a reverse transcription polymerase chain reaction PCR (RT-PCR). The PCR parameters were as follows: 95 °C for 3 min, followed by 35 cycles of 95 °C for 30 s, 58 °C for 30 s, and 72 °C for 1.5 min, and a final extension step of 72 °C for 10 min. All PCR products were separated by 1% agarose gel electrophoresis and sub-cloned into a pGEM-T Easy vector (Promega, Madison, WI, USA) for sequencing.

Conservative domains and sequence homology were predicted using blastp (https://blast.ncbi.nlm.nih.gov/Blast.cgi) (accessed on 1 September 2024) and mapped using IBS 1.0.1. The signal peptide was predicted with the SingnalP-5.0 server (https://services.healthtech.dtu.dk/services/SignalP-5.0/) (accessed on 9 September 2024). The molecular weight and isoelectric point were analyzed using ExPASy (http://web.expasy.org/compute_pi/) (accessed on 1 September 2024). A phylogenetic tree based on insect CDA sequences was constructed in MEGA 7 using the neighbor-joining method with 1000 bootstrap replicates and pairwise deletion [28].

### 2.3. Spatiotemporal Expression Analysis of TaCDA1

A total of 40 stage-specific samples were collected from 1st-, 2nd-, 3rd, and 4th-instar larvae (1L–4L), prepupae (PP), 1, 2, 3, 4, 5, 6, and 7 d old pupae (P1–7), and 1 d old adults (A1). These samples were promptly frozen in liquid nitrogen and then stored at −80 °C. In the tissue-specific experiment, various tissues, such as the head (HD), epidermis (EP), fat body (FB), Malpighian tube (MT), foregut (FG), and midgut (MG), were isolated from 80 4th-instar larvae. The larvae were dissected on ice under a stereomicroscope (Olympus, Tokyo, Japan) to maintain tissue freshness. Each sample was immediately placed in a 200 μL centrifuge tube containing RNAhold^®^ Reagent (TransGen, Beijing, China). All samples were collected four times to extract the total RNA and reverse transcribe cDNA. qPCR analyses were performed with a total reaction volume of 20 μL containing 10 μL of GoTaq qPCR Master Mix (Promega), 1 μL of each gene-specific primer, 1 μL of the cDNA template (500 ng/μL), and 7 μL of nuclease-free water. All primers used for qPCR were designed using Primer 3 (version 0.4.0) software (http://fokker.wi.mit.edu/primer3/) (accessed on 1 September 2024) and are listed in Table S1. The reaction conditions were as follows: 94 °C for 3 min, followed by 40 cycles of 94 °C for 30 s and 60 °C for 30 s. At the end of the cycling protocol, a melting curve from 60 to 95 °C was applied to verify the production of single PCR products. The *elongation factor 1-alpha* gene was used as an internal reference gene [29], and the 2^−ΔΔCt^ method was used to calculate the relative expression levels of *TaCDA1* [30].

### 2.4. Functional Analysis of TaCDA1 by RNAi

To explore the function of *TaCDA1* in *T. absoluta* development, ds*TaCDA1* and ds*GFP* were amplified with specific primers carrying the T7 RNA polymerase promoter sequence (Table S1). PCR products with verified sequences were employed as templates, and the dsRNAs were synthesized using the TranscriptAid T7 High Yield Transcription Kit (Thermo, Wilmington, DE, USA). For RNAi of pupae, 50 two-day-old pupae were injected with 200 nL dsRNA (2000 ng/μL) using a Nanoliter 2010 injector (WPI, Sarasota, FL, USA). To determine RNAi efficiency, 20 pupae from each group were collected 24, 48, and 72 h after dsRNA injection for qPCR. Additionally, the expression of seven chitin degradation genes, including *chitinase* (*TaCht1*, *TaCht2*, *TaCht5*, *TaCht7*, and *TaCht10)*, *imaginal disc growth factor* (*TaIDGF)*, and *β*-*N*-*acetylglucosaminidase* (*TaNAG1*), and four chitin synthesis genes including *trehalase* (*TaTre1* and *TeTre2)*, *UDP*-*N*-*acetylglucosamine pyrophosphorylase* (*TaUAP1)*, and *chitin synthase 1 (TaCHS1*) was measured. Phenotype changes were observed and photographed using a Keyence VHX-6000 stereomicroscope (Keyence, Osaka, Japan), and the survival rate within 8 d and weight of 30 individuals were recorded. For RNAi of 3rd-instar larvae, 50 individuals were transferred to a 1.5 mL centrifuge tube containing dsRNA solution (2000 ng/μL), soaked for 10 min, and moved to fresh tomato leaves for continuous feeding. To assess RNAi efficiency, 20 larvae from each group were collected 24, 48, and 72 h after soaking for qPCR. Phenotypic changes were observed using the Keyence VHX-6000 stereomicroscope, and the survival rate and abnormal death phenotype were recorded within 6 d. All experiments were performed in quadruplicate. Hematoxylin and eosin (H&E) staining was performed as previously described [31]. After soaking in dsRNA for 48 h, thirty insects were collected for cross slicing, fixation, staining, and elution and then photographed with an LSM 900 confocal laser-scanning microscope (Zeiss, Oberkochen, Germany).

### 2.5. The Impact of dsRNA Ingestion on Nesidiocoris tenuis

To determine whether ds*TaCDA1* had negative effects on non-target insects, *Nesidiocoris tenuis*, a predator of the tomato leaf miner, was used as a model organism for risk assessment. Briefly, a cotton ball was inserted into a sterile 1.5 mL centrifuge tube and soaked with 2.5 µL sucrose solution (0.5 M) + 2.5 µL ds*GFP* (2000 ng/μL) or 2.5 µL sucrose solution (0.5 M) + 2.5 µL ds*TaCDA1* (2000 ng/μL). The 3rd-instar nymphs of a single *N. tenuis* (*n* = 50 for each of the four replicates) was transferred into a 1.5 mL centrifuge tube. *NtCDA1* expression was detected after 48 h, and the survival rate was determined within 8 d.

### 2.6. Statistical Analysis

SPSS 20.0 (IBM Corp., Chicago, IL, USA) was utilized for statistical analysis. Expression data are presented as the mean ± SE (standard error). The Kaplan–Meier method was employed to analyze the survival rates, and the data were mapped using PRISM v6.01 (GraphPad, La Jolla, CA, USA). Significant differences among more than two samples were analyzed by one-way analysis of variance (ANOVA), and those between the two groups were compared using Student’s *t*-test.

## 3. Results

### 3.1. Identification of TaCDA1 in T. absoluta

**Figure 1:**
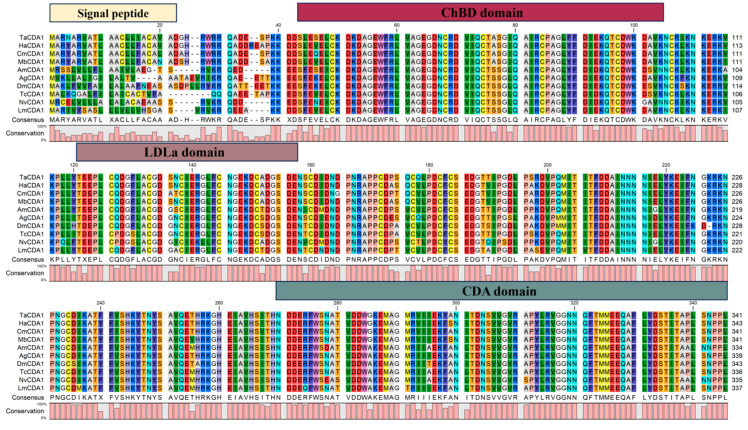
Multiple sequence alignment of *TaCDA1* from *Tuta absoluta* with CDA1 from other insects. Origin species of proteins and their GenBank accession numbers: TaCDA1: *T. absoluta*, KAJ2953570.1; HaCDA1: *Helicoverpa armigera*, XP_021189210.1; CmCDA1: *Cnaphalocrocis medinalis*, AJG44549.1; MbCDA1: *Mamestra brassicae*, AEI30868.1; AmCDA1: *Apis mellifera*, XP_391915.1; AgCDA1: *Anopheles arabiensis*, XP_040165945.1; DmCDA1: *Drosophila melanogaster*, NP_001262062; TcCDA1: *Tribolium castaneum*, NP_001095946.1; NvCDA1: *Nasonia vitripennis*, XP_001604765.1; LmCDA1: *Locusta migratoria*, ANA57443. The yellow box represents the signal peptide, the red box represents the ChBD domain, the brown box represents the LDLa domain, and the green box represents the CDA domain.

The full-length ORF of *TaCDA1* was 1620 bp and encoded 539 amino acids. The theoretical protein molecular weight was 61.29 kDa, and the isoelectric point was 5.03. Sequence alignment revealed that TaCDA1 had a high resemblance to CDA1 in other insects, featuring three conserved domains, namely the chitin-binding domain (ChBD, amino acids 43–104), the low-density lipoprotein receptor class a domain (LDLa, amino acids 121–158), and the deacetylase catalytic domain (CDA, amino acids 274–340), suggesting that the protein encoded by TaCDA1 was a typical class I CDA protein (Figure 1).

**Figure 2:**
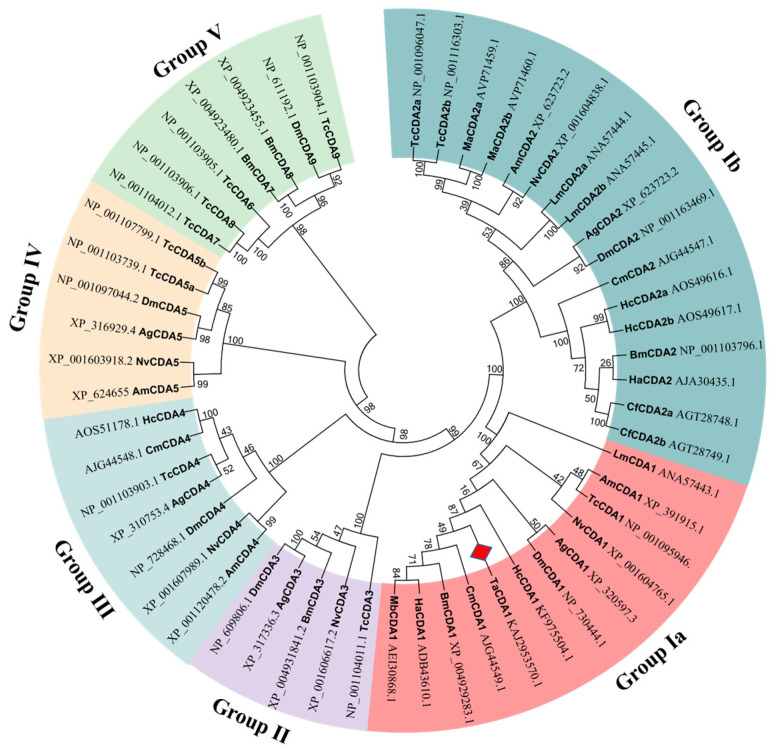
Phylogenetic tree of CDA proteins from *Tuta absoluta* and other insect species. TaCDA1 is annotated with a red diamond. *Anopheles gambiae*: AgCDA1, AgCDA2, AgCDA3, AgCDA4, AgCDA5; *Apis mellifera*: AmCDA1, AmCDA2, AmCDA4, AmCDA5; *Drosophila melanogaster*: DmCDA1, DmCDA2, DmCDA3, DmCDA4, DmCDA5, DmCDA9; *Tribolium castaneum*: TcCDA9, TcCDA8, TcCDA7, TcCDA6, TcCDA5b, TcCDA5a, TcCDA4, TcCDA3 TcCDA2a, TcCDA2b, TcCDA1; *Nasonia vitripennis*: NvCDA1, NvCDA2, NvCDA3, NvCDA4, NvCDA5; *Cnaphalocrocis medinalis*: CmCDA1, CmCDA2, CmCDA4; *Bombyx mori*: BmCDA1, BmCDA2, BmCDA3, BmCDA7, BmCDA8; *Helicoverpa armigera*: HaCDA1, HaCDA2; *Locusta migratoria*: LmCDA1, LmCDA2a, LmCDA2b; *Mamestra brassicae*: MbCDA1, MbCDA2a, MbCDA2b; *Choristoneura fumiferana*: CfCDA2a, CfCDA2b; *Hyphantria cunea*: HcCDA1, HcCDA2a, HcCDA2b, HcCDA4.

Homology analysis demonstrated that TaCDA1 had 98.14, 96.47, and 95.92% identity with CDA1 of *Phthorimaea operculella* (KAI5641539.1), *Ostrinia furnacalis* (XP_028176771.1), and *Cydia fagiglandana* (XP_063376228.1), respectively. Phylogenetic analysis revealed that insect CDAs grouped into five independent clusters (I–V), and Group I CDAs were further divided into two subgroups (Group CDA-Ia and Group CDA-Ib). TaCDA1 was most closely associated with *Cnaphalocrocis medinalis* CDA1 and belonged to the Group Ia CDAs (Figure 2).

### 3.2. Spatiotemporal Expression Analysis

**Figure 3:**
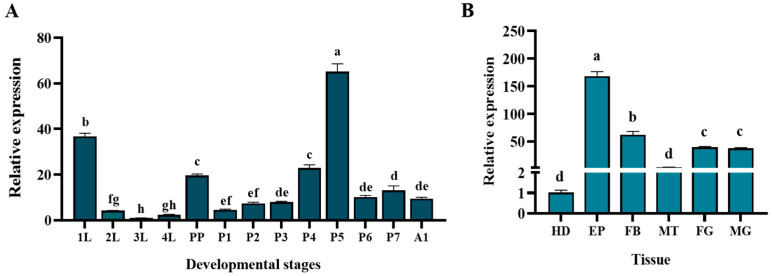
Expression patterns of *TaCDA1* at different developmental stages and in different tissues of *Tuta absoluta*. Expression pattern of *TaCDA1* at different developmental stages in *T. absoluta* (**A**). Expression pattern of *TaCDA1* in different tissues of *T. absoluta* (**B**). 1L–4L: 1st-, 2nd-, 3rd-, and 4th-instar larvae; PP: prepupa; P1–7: 1-, 2-, 3-, 4-, 5-, 6-, and 7 d old pupae; A1: 1 d old adult; HD: head; FB: fat body; EP: epidermis; FG: foregut; MT: Malpighian tube; MG: midgut. Different lowercase letters indicate significant differences between different developmental stages and tissues (*p* < 0.05).

The expression patterns of the *TaCDA1* gene in *T. absoluta* during different development stages indicated that it peaked at the 5 d old pupa stage, followed by the 1st-instar larvae and prepupae, and was lowest in the 3rd-instar larvae (*F*(_12,39_) = 198.199, *df* = 12, *p* < 0.0001) (Figure 3A). *TaCDA1* was expressed in all examined tissues, and the expression level was highest in the epidermis, being 164.82 times that in the head (*F*(_5,18_) = 203.565, *df* = 5, *p* < 0.0001) (Figure 3B).

### 3.3. Effects of Suppressing TaCDA1 on the Pupal–Adult Transition

**Figure 4:**
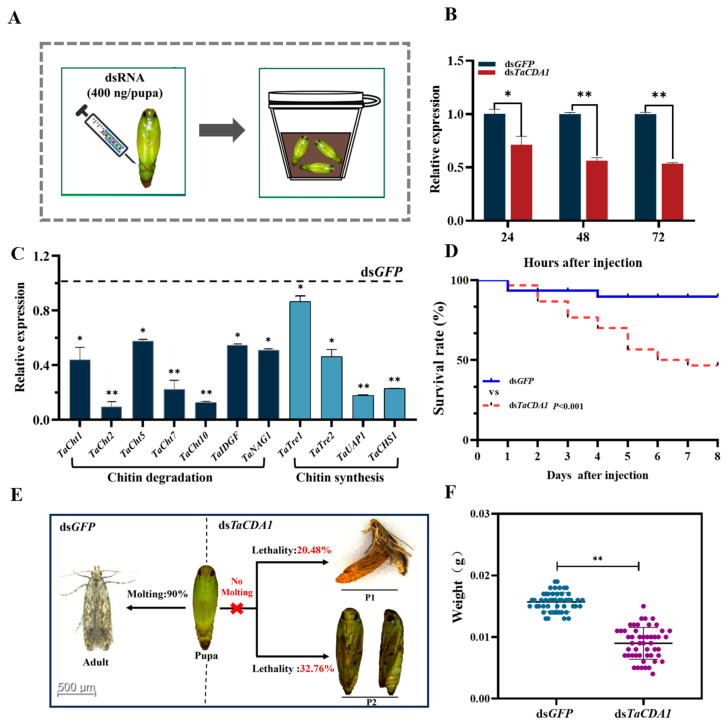
Effect of *TaCDA1* knockdown on pupal–adult metamorphosis in *Tuta absoluta*. Design of injecting pupae with dsRNA. Each 2 d old pupa was injected with dsRNA, and then placed in Petri dish with soil for phenotypic observation (**A**). Relative expression levels of *TaCDA1* at 24, 48, and 72 h after *TaCDA1* or *GFP* dsRNA injection (**B**). Expression of chitin metabolism pathway genes after silencing *TaCDA1* (**C**). Kaplan–Meier survival curves of *T. absoluta* pupae after *TaCDA1* or *GFP* dsRNA injection (**D**). Representative phenotypes of pupae after *TaCDA1* or *GFP* dsRNA injection (**E**). Pupa weight in *T. absoluta* after silencing *TaCDA1* (**F**). Asterisks indicate significant differences (* *p* < 0.05, ** *p* < 0.01).

RNAi mediated through injection was utilized to investigate the function of the pupal–adult stage transition in *T. absoluta* (Figure 4A). The mRNA expression level of *TaCDA1* decreased by 28.92, 43.68, and 46.51% at 24, 48, and 72 h after injection with ds*TaCDA1*, respectively (24 h: *t* = 4.33, *p* = 0.017709; 48 h: *t* = 13.62, *p* < 0.0001; 72 h: *t* = 24.93, *p* < 0.0001) (Figure 4B). Compared to the control, the expression of genes related to chitin degradation (*TaCht1*, *TaCht2*, *TaCht5*, *TaCht7*, *TaCht10*, *TaIDGF*, and *TaNAG1*) and chitin synthesis (*TaTre1*, *TeTre2*, *TaUAP1*, and *TaCHS1*) decreased significantly after injection with ds*TaCDA1* (Figure 4C). After injection with ds*GFP*, 90.00% of the test insects in the control completed the transformation from pupae to adults (Figure 4D). However, 8 d after injection with ds*TaCDA1*, the mortality rate of the test insects reached 53.24% (*p* = 0.002) (Figure 4D). The death phenotype was recorded as follows: P1, the pupa appeared hollow and shriveled, could not shed its skin, and died; and P2, the individuals could not shed their old epidermis and died during eclosion (Figure 4E). The weight of the two groups of test insects was examined, and silencing *TaCDA1* led to a significant reduction of 0.007 g (*F* = 12.098, *t* = 13.681, *p* < 0.001) in pupa weight (Figure 4F).

### 3.4. Effects of Suppressing TaCDA1 on Larval–Pupal Transition

**Figure 5:**
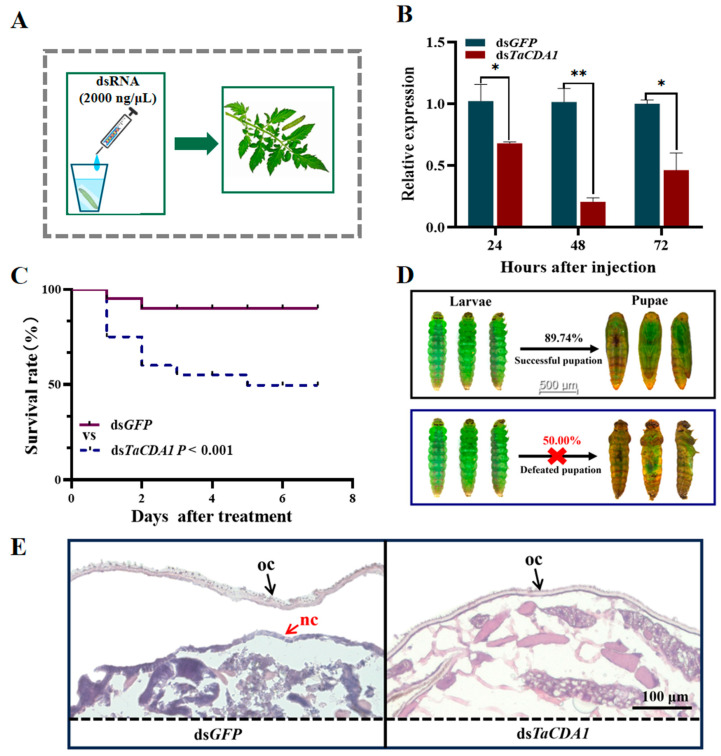
Effect of *TaCDA1* knockdown on larval–pupal transition in *Tuta absoluta*. Design of soaking 3rd-instar larvae with dsRNA. Each 3rd-instar larva was soaked in dsRNA solution for 10 min, and then transferred to fresh leaves for feeding (**A**). Relative expression levels of *TaCDA1* 24, 48, and 72 h after *TaCDA1* or *GFP* dsRNA injection in the larvae (**B**). Kaplan–Meier survival curves of *T. absoluta* larvae after *GFP* or *TaCDA1* dsRNA injection (**C**). Representative phenotypes of larvae after *GFP* or TaCDA1 dsRNA injection (**D**). Hematoxylin and eosin staining of the epidermis after *GFP* or *TaCDA1* dsRNA injection (**E**). nc: new cuticle; oc: old cuticle. “*”: *p* < 0.05; “**”: *p* < 0.01.

RNAi mediated through soaking was employed to study the function of the larval–pupal stage transition in *T. absoluta* (Figure 5A). The expression level of *TaCDA1* significantly decreased by 34.21, 80.86, and 53.68% at 24, 48, and 72 h after ds*TaCDA1* injection, respectively, compared to the control (24 h: *t* = 2.528, *p* = 0.044805; 48 h: *t* = 7.027, *p* < 0.0001; 72 h: *t* = 3.786, *p* = 0.00912) (Figure 5B). After injection with ds*GFP*, 89.74% of the test insects in the control group completed the transformation from larvae to pupae (Figure 5C and D). At 7 d after injection with ds*TaCDA1*, the mortality rate of the test insects reached 50.00% (*p* = 0.0084) (Figure 5C), with deaths occurring during pupation without the formation of a new epidermis (Figure 5D). H&E staining demonstrated that the old cuticle of the ds*GFP* injection group was digested and successfully separated from the new epidermis beneath (Figure 5E). However, the insects in the ds*TaCDA1* injection group did not form new cuticles or shed their old cuticles (Figure 5E).

### 3.5. Effects of Oral Feeding of dsTaCDA1 in N. tenuis

**Figure 6:**
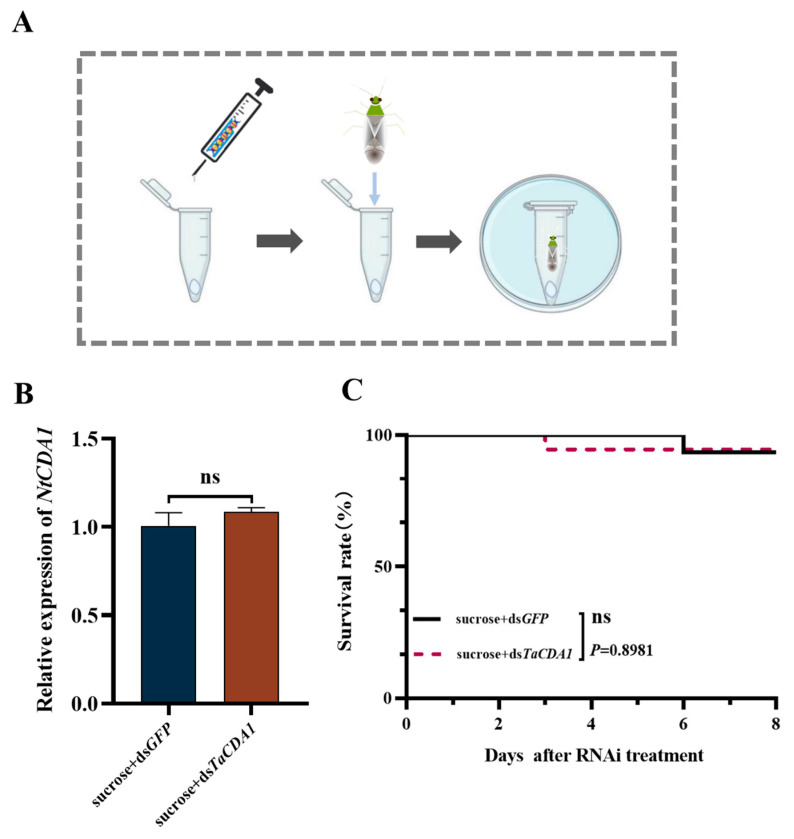
Impact of *Tuta absoluta* dsRNA intake on *Nesidiocoris tenuis*. Experimental setup for the oral delivery of dsRNA via sucrose (**A**). Relative expression levels of *NtCDA1* 48 h after sucrose + *TaCDA1* or sucrose + *GFP* dsRNA feeding in *N. tenuis* (**B**). Effect of non-homologous dsRNA on the survival rate of *N. tenuis* (**C**). ns: not significance.

The qPCR results revealed that oral feeding with the ds*TaCDA1* + sucrose solution had no notable impact on *NtCDA1* compared to the ds*GFP* + sucrose solution oral feeding group (*t* = 1.004, *p* = 0.8981) (Figure 6B). Similarly, there were no significant effects on mortality when feeding dsRNA between the two groups (Figure 6C).

## 4. Discussion

Chitin deacetylases have an essential role in chitin degradation among insects and have been a research hotspot since their discovery [32,33]. With the advancement of research, a growing number of insect CDAs have been successfully identified, such as those in Hemiptera [34], Coleoptera [9], Orthoptera [35], Lepidoptera [36], and Diptera [13]. In this study, we cloned and identified the *CDA1* gene in *T. absoluta* and found that it belonged to Group Ia. In this study, *TaCDA1* of *T. absoluta* showed significant temporal and spatial differences, and it was expressed at all evaluated developmental stages. *TaCDA1* expression was highest in the 5 d old pupa, followed by the 1st-instar larvae, and was lowest during the adult stage. The 1st-instar larvae and 5 d old pupa are key stages in the growth and development transformation of *T. absoluta* from egg to larva and from pupae to adult, respectively. This expression pattern is consistent with the *CDA1* expression trend in various insects. For example, *SfCDA1* of *S. furcifera* was highly expressed in 1 d old 1st-instar nymphs and 3 d old 5th-instar nymphs [5]. In *Locusta migratoria*, *LmCDA1* expression peaked in early and late nymphs [35]. In *Lasioderma serricorne*, *CDA1* was highly expressed in late pupae and late larvae, and the lowest expression was observed during the adult stage [37]. *CDA1* expression in *Hyphantria cunea* was highest in 6th-instar larvae [38]. A tissue specificity study showed that *TaCDA1* was highly expressed in the epidermis, which is consistent among most insects, such as *SfCDA1* in *S. furcifer*a [5], *NiCDA1* in *N. lugens* [8], *HpCDA1* in *H. parallela* [11], *HvCDA1* in *Heortia vitessoides* [6], and *HcCDA1* in *H. cunea* [38]. These results suggest that *TaCDA1* may be closely related to the growth and development of *T. absoluta*.

The pupal stage is a crucial period for insects to transition from larvae to adults. During this stage, significant physiological changes occur inside the pupa, including comprehensive adjustments in body shape, internal and external organ structure, and functional differentiation [39]. This transformation ensures the maturation and reproductive ability of insects, with the tissues and organs undergoing reorganization and regeneration [39]. Degradation of the old epidermis and formation of the new epidermis are extremely important for structural support and mobility in adult insects and provide protection for the organisms inside [32]. In the current study, after silencing *TaCDA1* in the 2-day-old pupae of *T. absoluta*, the test insects died and could not emerge. This phenomenon also occurs in most other insects, such as with *LsCDA1* in *L. serricorn*e [37], *SfCDA1* in *S. furcifera* [5], *HcCDA1* in *H. cunea* [38], and *HpCDA1* in *H. parallela* [11]. Furthermore, silencing *LmCDA1* in *L. migratoria* increased its sensitivity to the pesticides malathion and chlorpyrifos and fungus *Metarhizium anisopliae*. Chitin is the main component of the epidermis; it tightly binds to cuticle proteins through hydrogen bonds, making the epidermis lightweight and tough. Thus, chitin degradation and synthesis are key processes during the transformation of epidermis [40]. CDAs catalyze the *N*-deacetylation of chitin to produce chitosan, which is an enzyme widely distributed in bacteria [41], fungi [42], and insects [43]. In this study, inhibiting *TaCDA1* expression also reduced the expression of genes related to chitin synthesis and degradation. In *L. serricorne*, knocking down *LsCDA1* led to molting defects, and the expression of *LsTRE1*, *LsUAP1*, and *LsCHS1* was significantly decreased. Similarly, silencing of *LdCDA1* in *L*. *decemlineata* significantly reduced the chitin amounts and the expression of five chitin synthesis pathway genes [44]. These results collectively indicate that the *TaCDA1* gene is involved in chitin synthesis and degradation in *T. absoluta* and can be used as a potential target for blocking insect development.

Insect CDAs function in organ formation and molting, and CDAs do not exist in humans or plants. Therefore, substances targeting CDAs usually have high interference in insect growth and development and high specificity [45]. It is a valuable way to develop novel and effective insect control strategies [46]. Insects have different CDAs with distinct tissue specificity to regulate development and survival. Genes encoding CDAs belonging to Group I are usually highly expressed before transformation the during egg–larva, larva–larva, larva–pupa, and pupa–adult transitions, and this kind of gene is mainly expressed in the integument [11,17]. *TaCDA1* expression conforms to this pattern, indicating that its function is closely related to the transformation of the epidermis. RNAi technology provides an ideal tool to disturb the function of CDAs at the molecular level. In *H. vitessoides*, post-injection of dsRNA targeting *HvCDA1* caused 33% mortality during the larval–pupal transition and 77% mortality during the pupal–adult transition [6]. RNAi of *SfCDA* genes in *S. furcifera* influenced the transcripts of chitin synthase and trehalase, resulting in high mortality during nymph–adult molting [5]. Injection of dsRNA targeting *HcCDA1* in *H. cunea* resulted in larval–pupal molting difficulty and produced high larval mortality [38]. In *T. absoluta*, soaking with specific dsRNA of 3rd-instar larvae led to an increase in the mortality rate from the larval to the pupal stage, and the affected test insects were unable to form a new cuticle. These studies have demonstrated that with RNAi technology, targeting Group I CDAs is lethal to insects from various orders, disturbing the molting process during larval–pupal and pupal–adult transitions. In addition to molting, CDAs also function in arthropod locomotion and muscle attachment. Depletion of *CDAs* in *T*. *castaneum* led to the breakage of the internal tendon cuticle at the femur–tibia joint, the detachment of muscles from both internal and external tendon cells, and locomotion defects [47].

RNAi technology shows great potential in the fields of insect research and pest control. However, just like any emerging technology, a comprehensive assessment of its safety is of vital importance. In this study, oral administration of ds*TaCDA1* to *N. tenuis* did not affect the expression of its target gene and mortality, indicating that the dsRNA specific to *TaCDA1* was safe for non-target insects. In addition, with dsRNA as an active compound, the most important point that makes RNAi control successful is the efficient delivery of dsRNA to pests. However, there are many natural obstacles to the delivery of naked dsRNA. The main pathways for pesticide molecules to enter insect bodies are through penetration into the epidermis by direct application on the insect body and crossing the intestine by feeding [48]. Unfortunately, it is very hard for naked dsRNA to penetrate the epidermis or intestinal wall of insects, and naked dsRNA can be easily decomposed by enzymes in the environment or inside the intestine [48]. Although, in certain successful studies, naked dsRNA worked well by spraying, the success was inseparable from the physiological characteristics of the insects (e.g., insects of Coleoptera are quite sensitive to RNAi) [49]. In this case, RNAi primarily worked only through injection, and this study is no exception. In recent years, researchers have also been committed to determining an efficient method to deliver dsRNA, and using nanomaterials as carriers seems like a solution to this question. Nano-delivery systems have shown the ability to increase the stability of dsRNA in complex environments and help dsRNA to cross various biological and physical barriers, such as insect epidermal and intestinal cells [50]. Thus, it is predictable that for RNAi control in the field, spraying dsRNAs with nano-carriers is an effective way to achieve good results. Genetic modification is an alternative way to make plants express large amounts of dsRNA and kill pests through feeding [51].

## 5. Conclusions

In summary, we identified and cloned *TaCDA1* in the invasive insect species *T. absoluta* and explored its roles during molting and metamorphosis. Suppression of *TaCDA1* expression in *T. absoluta* pupae significantly disrupted development during the pupal–adult transition, increasing mortality and reducing pupal weight by affecting chitin metabolism. The oral administration of ds*TaCDA1* resulted in elevated mortality rates and did not affect *NtCDA1* expression or mortality rates in the non-target insect *N. tenuis*. This study indicates that *TaCDA*1 can be used as a target for pest control in *T. absoluta* through genetic manipulation.

## Data Availability

Data are contained within the article or Supplementary Materials.

## Supplementary Materials

The following supporting information can be downloaded at https://www.mdpi.com/article/10.3390/insects15110838/s1: Table S1: Primers used in this study.
