# Supplementary material for: Chitin Deacetylase 1 Gene as an Optimal RNAi-Based Target for Controlling the Tomato Leaf Miner Tuta absoluta
Source: Insects. 2024 Oct 25;15(11):838. doi: 10.3390/insects15110838 (PMC11595049; doi:10.3390/insects15110838)
Supplement: Supplementary file 1 [file insects-15-00838-s001.zip › insects-3245885-supplementary.pdf]

**Table S1. Primers used in this study.**

| Gene name | Forward primer (5′-3′) | Reverse primer (5′-3′) | Application primers |
|---|---|---|---|
| *TaCDA1* | CAACGCCCGTGTCGCTAC | AGAGGTCAGTTTGCAAGAGT | ORF confirmation |
| *TaCDA1* | AAGAAAGACGACAGCCTGGA | CTTCACTTTGCGCTCCTTGT | qPCR analysis |
| *TaEF1α* | CCTGGGCACAGAGATTTCAT | GATCAGCTGCTTGACACCAA | |
| *TaCht1* | GTCCGATAAGAAGGGGCAGA | ATTCCGAGAAGCCACATCCT | |
| *TaCht2* | GTTTTGACCGTGGCTTCTGT | CATGTGGATGAGGGTCAGGT | |
| *TaCht5* | AGAAGAAGGGCTATCTGGGC | AGGGTCAGGAGTTGTGGATG | |
| *TaCht7* | CTCAGCGGGTACAAAGTTCG | GTGGTGCTTTCTCTCTCCCT | |
| *TaCht10* | CCGGAAATGGATTAGGTGCG | CCTTGTGGCGGATCATGAAG | |
| *TaIDGF* | CCGGAAATGGATTAGGTGCG | CCTTGTGGCGGATCATGAAG | |
| *TaTre1* | GGTTAGACACGCAACGGATC | CTCCTGTTGCCGTTCTGATG | |
| *TaTre2* | CGCGCCGATATTATTGAGGG | CTCCTTCCAAACACGTAGCG | |
| *TaUAP* | AACCGCTCGAGATGATTTGC | ACCGTTACCATTCAGTCCGT | |
| *TaChs* | TTGTCGTCCATGAGAGCCTT | CCTTGTGGCGGATCATGAAG | |
| *NtCDA1* | CCTATGACGATTGGCTTGCC | GGCAGTGATTGAAGCATCGT | |
| *NtATPB* | CATACGCCAAGGGAGGTAAA | CTGGGTGAAACGGAAAATGT | |
| *TaCDA1* | **TAATACGACTCACTATAGGG**ACCCGTCGCAATGTCAGTT | **TAATACGACTCACTATAGGG**TTGAGCAAGAGTCTACCATA | dsRNA synthesis |
| *GFP* | **TAATACGACTCACTATAGGG**TACAAGACGCGTGCTGAAGT | **TAATACGACTCACTATAGGG**CAATGTTGTGGCGAATTTTG | |

The sequence in bold at the 5’end of the primer is the T7 promoter sequence.
